# The bacterial gut microbiome of probiotic-treated very-preterm infants: changes from admission to discharge

**DOI:** 10.1038/s41390-021-01738-6

**Published:** 2021-10-07

**Authors:** Jacob A. F. Westaway, Roger Huerlimann, Yoga Kandasamy, Catherine M. Miller, Robert Norton, Kyran M. Staunton, David Watson, Donna Rudd

**Affiliations:** 1 College of Public Health, Medical and Veterinary Science, James Cook University, 1/14-88 McGregor Road, Smithfield, QLD 4878 Australia; 2 Centre for Tropical Bioinformatics and Molecular Biology, James Cook University, 1 James Cook Drive, Douglas, QLD 4811 Australia; 3 Marine Climate Change Unit, Okinawa Institute of Science and Technology (OIST), 1919-1 Tancha, Onna-son Okinawa, 904-0495 Japan; 4 College of Science and Engineering, James Cook University, 1 James Cook Drive, Douglas, QLD 4811 Australia; 5 College of Public Health, Medical and Veterinary Science, James Cook University, 1 James Cook Drive, Douglas, QLD 4811 Australia; 6 Department of neonatology, Townsville University Hospital, 100 Angus Smith Drive, Douglas, QLD 4814 Australia; 7 Australian Institute for Tropical Health and Medicine, James Cook University, 1/14-88 McGregor Road, Smithfield, QLD 4878 Australia; 8 Department of Microbiology, Pathology Queensland, 100 Angus Smith Drive, Douglas, QLD 4814 Australia; 9 Department of Maternal-Fetal Medicine, Townsville University Hospital, 100 Angus Smith Drive, Douglas, 4814 Australia

## Abstract

**Background:**

Preterm birth is associated with the development of acute and chronic disease, potentially, through the disruption of normal gut microbiome development. Probiotics may correct for microbial imbalances and mitigate disease risk. Here, we used amplicon sequencing to characterise the gut microbiome of probiotic-treated premature infants. We aimed to identify and understand variation in bacterial gut flora from admission to discharge and in association with clinical variables.

**Methods:**

Infants born <32 weeks gestation and <1500 g, and who received probiotic treatment, were recruited in North Queensland Australia. Meconium and faecal samples were collected at admission and discharge. All samples underwent 16S rRNA short amplicon sequencing, and subsequently, a combination of univariate and multivariate analyses.

**Results:**

71 admission and 63 discharge samples were collected. Univariate analyses showed significant changes in the gut flora from admission to discharge. Mixed-effects modelling showed significantly lower alpha diversity in infants diagnosed with either sepsis or retinopathy of prematurity (ROP) and those fed formula. In addition, chorioamnionitis, preeclampsia, sepsis, necrotising enterocolitis and ROP were also all associated with the differential abundance of several taxa.

**Conclusions:**

The lower microbial diversity seen in infants with diagnosed disorders or formula-fed, as well as differing abundances of several taxa across multiple variables, highlights the role of the microbiome in the development of health and disease. This study supports the need for promoting healthy microbiome development in preterm neonates.

**Impact:**

- Low diversity and differing taxonomic abundances in preterm gut microbiota demonstrated in formula-fed infants and those identified with postnatal conditions, as well as differences in taxonomy associated with preeclampsia and chorioamnionitis, reinforcing the association of the microbiome composition changes due to maternal and infant disease.
- The largest study exploring an association between the preterm infant microbiome and ROP.
- A novel association between the preterm infant gut microbiome and preeclampsia in a unique cohort of very-premature probiotic-supplemented infants.

## Introduction

It is well known that preterm birth leads to retarded gut microbiome development and increased risk of acute and chronic disease in infants and adults.^1^ The gut microbiome composition of preterm infants differs significantly from those born full-term, and is characterised by lower diversity^2,3^ and high interindividual variation.^4–6^ In addition, despite high variability, preterm infants typically have fewer commensals like *Bifidobacterium*^4,6^ and *Lactobacillus*,^4,7^ and more potential pathogens like *Klebsiella pneumoniae*^8^ and *Clostridium difficile*.^6^ However, the gut microbiome is dynamic and changes significantly over time.^9^ Although reduced levels of common commensal organisms and diversity can persist for months,^10,11^ maybe years,^12^ choreographed abrupt changes in composition^13,14^ and increases in diversity^11^ mean that eventually the preterm gut microbiome composition becomes more similar to that of full-term infants.

Shifts in composition and organism dominance result from environmental changes and major colonising events. Colonisation occurs via different routes and is influenced by several factors, including delivery and diet. Delivery is the first major colonising event, contributing significantly to differences between individuals,^15,16^ including higher abundances of vaginally derived microbes in those born vaginally. This includes *Bacteroides* and *Lactobacillus*.^15,17^ In contrast, caesarean-born infants acquire greater abundances of skin-dwelling microbes like *Staphylococcus*.^11,15^ As for diet, breastmilk and formula also produce distinct microbial communities^9,18^ due to the presence of both microbes and human-made oligosaccharides (HMOs) in breastmilk.^19^ Although maternal skin and vaginal microbes colonise infants during birth and feeding, these microbes may only be transient with maternal gut microbes, passed through birth or lactation proving to be more persistent.^20^

As much of the microbial inoculation occurs through maternal–infant exchange, maternal health and medical interventions can also influence the developing infant microbiome. Interventions such as antibiotics^21^ and diseases like chorioamnionitis,^16^ a bacterial infection occurring before or during labour, have been previously shown to influence the infant microbiome. Thus, other maternal microbiome-altering diseases, like type 2 diabetes^22^ and preeclampsia,^23^ a pregnancy disorder characterised by high blood pressure, could also disrupt the infant microbiome. The resulting irregular infant microbiome could have severe consequences for infant health and development.^24^

Disrupted microbial colonisation puts preterm infants at a high risk of acute infection,^25,26^ chronic disease^27,28^ and developmental abnormalities.^29,30^ The increased risk of disease is a consequence of the breakdown in the symbiotic relationship between infants and colonising microbes, with delayed colonisation of commensal microbes contributing to intolerances to normal flora.^31,32^ Additive to this is an imbalance between commensals and pathogens that may induce intestinal inflammation and cytokine production.^33^ These microbial imbalances contribute to higher rates of acute diseases like necrotising enterocolitis (NEC) and sepsis, chronic diseases like asthma^34^ and potentially, developmental disorders like retinopathy of prematurity (ROP)^35^ in preterm infants. This disproportionate burden of the disease leads to microbiome-altering treatment with antibiotics, a staple in preterm neonatal care and probiotics, an emerging preventative strategy. Antibiotics can disrupt microbial acquisition, resulting in reduced diversity and altered bacterial profiles,^36^ whilst probiotics have been shown to promote the growth of commensal microbes and increases in diversity,^37–39^ as well as reducing disease incidence.^40^

As probiotic treatment is now common for the most premature of infants, this prospective observational study using 16S ribosomal RNA (rRNA) high-throughput analysis of faecal and meconium samples aimed to characterise the bacterial gut microbiome of probiotic preterm infants. Specifically, we set out to characterise changes in a probiotic-supplemented cohort of preterm infants from admission to discharge, and to examine the impact of several key variables on the microbiome. This includes assessing the reproducibility of past findings and exploring new potential associations through multivariant analyses.

## Methods

### Study population

16S rRNA high-throughput sequencing was used to characterise the bacterial microbiome, down to genus, of infants receiving probiotic treatment and born into the Townsville Hospital and Health Service’s (THHS) Neonatal Intensive Care Unit (NICU). The THHS NICU is the only level six tertiary referral unit outside southeast Queensland, Australia. Thus, all babies being born at <29 gestation weeks in North Queensland (NQLD) are referred here. NQLD is affected disproportionately by preterm birth, with the North West experiencing the highest rate (12%) of preterm births,^41^ and the Torres and Cape the highest proportion (11.7%) of low birth weight infants.^41^ NQLD also has a large indigenous population, whose infants are more likely to be born prematurely (13%) and represent one out of ten premature births in Queensland.^41^ When considering the increasing prevalence of premature birth in the NQLD, 5% over the past decade,^41^ the burden that preterm birth places on NQLD families and the healthcare system is significant.

### Study design and ethics

Ethics was obtained from the Human Research Ethics Committee from the THHS, and recruitment commenced in October of 2017 and continued until October of 2018. Inclusion criteria were infants born <32 weeks’ gestation and admitted to the NICU at the THHS. The exclusion criteria were no parental consent, gestational age of >32 weeks and contraindication to enteral feeds. One capsule of the probiotic Infloran®,^42^ containing *Lactobacillus acidophilus* (1 × 10^9^ CFU) and *Lactobacillus bifidus* (*Bifidobacterium bifidum*) (1 × 10^9^ CFU), is administered via enteral feeds to all infants born <32 weeks gestations and <1500 g at the THHS NICU on a daily basis. Infloran® treatment is commenced on the first day of feeding and ceased once the infant is >34–36 weeks gestation. Recruitment was conducted by a neonatal nurse/research assistant who works at the NICU, and sample collection was carried out by NICU nurses using collection kits (biohazard bag, sterile swab and storage container). Collection occurred at admission (meconium) and just prior to discharge (stool). After collection, samples were sent via a pneumatic tube system to Pathology Queensland and stored at −80 °C. Clinical information was also collected for downstream analysis. This included both maternal data—antenatal antibiotics, antenatal infections (clinically diagnosed), chorioamnionitis (clinically diagnosed), prolonged membrane rupture (clinically diagnosed), preeclampsia (clinically diagnosed) and diabetes (type 1 or 2, self-reported), and infant data—sex, mode of delivery (vaginal birth versus Caesarean section), diet, gestation at birth and collection, NEC (stage 2 or greater), sepsis (confirmed through culture), days and timing of antibiotics, death, ROP (stage 1 or greater), birth weight, nursery discharge weight and date of birth. A summary of these data can be found in Table 1.

**Table 1:** Overview of the demographic data for the cohort.

Variables	Levels	Count		Percentage (%)
Categorical variables				
Sex	Male	60		44.8
	Female	74		55.2
Diet	Formula	40		29.9
	Breastmilk	64		47.8
	Formula and breastmilk	30		22.4
Delivery	Vaginal	45		33.6
	Caesarean	89		66.4
NEC	Yes	12		9.0
	No	122		91.0
Sepsis	Yes	8		6.0
	No	126		94.0
Died	Yes	7		5.2
	No	127		94.8
Antenatal antibiotics	Yes	90		67.2
	No	44		32.8
Neonatal antibiotics	Yes	126		94.0
	No	8		6.0
Chorioamnionitis	Yes	58		43.3
	No	76		56.7
Preeclampsia	Yes	20		14.9
	No	114		85.1
Maternal diabetes	Yes	24		17.9
	No	110		82.1
Continuous variables	Mean/median			
Gestational age at birth	28.3/28.1 weeks			
Gestational age at sample collection	Admission		29.3/29.3	
	Discharge		35.3/35.9	
Days on antibiotics prior to sample collection	Admission		3.3/3	
	Discharge		9.4/5	
Weight at birth	1193/1086 g			
Weight at discharge	2448/2425 g			

*NEC* necrotising enterocolitis, *ROP* retinopathy of prematurity.

### Sequencing and bioinformatics

In brief, the protocol used in this study included sample storage at −80 °C,^43^ an extraction kit that includes mechanical lysis,^44^ use of the Illumina MiSeq platform,^45^ targeting of the V3/V4 regions^46^ and use of the SILVA reference database.^46^

DNA extraction was conducted using the Bioline ISOLATE Faecal DNA Kit,^47^ with modifications made in consultation with the manufacturer to optimise DNA yield. This included increased beta-mercaptoethanol (from 0.5 to 1% to increase DNA solubility and reduce secondary structure formation), the addition of an extra wash step (to improve purity) and decreased elution buffer volume (to increase final DNA concentration). For library preparation, we followed the Illumina metagenomics library preparation protocol,^48^ using the Index Kit v2 C,^49^ along with Platinum™ SuperFi™ PCR Master Mix.^50^ The MiSeq Reagent Kit V3^49^ was used in combination with the Illumina MiSeq System, targeting the V3 and V4 regions with the S-D-Bact-0431-b-S-17/S-D-Bact-0785-a-A-21785F primer combination for sequencing.

Pre-analytical bioinformatics was conducted in *R Studio* Version 3.6.1^51^ with a pipeline adapted from *Workflow for Microbiome Data Analysis: from raw reads to community analyses*,^52^ which along with the subsequent analyses can be found under Supplementary Material. *DADA2*^53^ was used for quality filtering and trimming, demultiplexing, denoising and taxonomic assignment (with the SILVA Database), and the *microDecon* package^54^ was used to remove homogenous contamination from samples using six blanks originating in extraction.

### Statistical analysis

#### Exploring changes in composition and diversity from admission to discharge

For statistical analysis, a phyloseq object was created using the package *Phyloseq*,^55^ with taxa filtered by prevalence (threshold = 0.01) and agglomerated at the genus level. The data were then explored through principal coordinate analysis (PCoA) plots using a Bray–Curtis dissimilarity matrix created from normalised (Total Sum Scaling) non-agglomerated data. Permutational analysis of variance (PERMANOVA) was then conducted for community-level comparisons between admission and discharge samples to observe group-level differences based on the Bray–Curtis dissimilarity matrix, using the *adnois()* function of the package *Vegan*.^56^ Alpha-diversity indices, Shannon index and Observed (richness) were then calculated on filtered, non-agglomerated data, and a comparison was made between admission and discharge samples using a Wilcoxon’s rank-sum test, with adjusted *p* values accounting for false discovery rate using the Benjamini–Hochberg procedure.^57^ To identify individual microbes whose abundance changed significantly from admission to discharge, data that were filtered and agglomerated at the genus level, but not transformed, were then normalised and modelled (negative binomial) with *DESeq2*.^58^ A Wald test with the Benjamini–Hochberg multiple inference correction was then performed to determine significant differentially abundant taxa.

### Exploring the effect of clinical variables on alpha diversity and taxonomic abundance

Lastly, associations between several clinical variables and community structure were explored. The relationship between clinical variables and both Shannon diversity and taxonomic abundance was assessed using multivariant linear regression models. For exploring the relationship with Shannon diversity, a mixed-effects linear regression model was created using the package *lme4*,^59^ with a gaussian distribution and using the restricted maximum-likelihood estimation. Continuous predictors were scaled and centered to avoid convergence issues and multicollinearity assessed using the *AED* package.^60^ Collinear variables were removed from the model. Thirteen predictors: mode of delivery, feeding type, gestation, antenatal antibiotics, antenatal infections, NEC, sepsis, chorioamnionitis, neonatal antibiotics, death, prolonged membrane rupture, preeclampsia, diabetes and ROP were included in the initial model. To control for high amounts of interindividual variation in the microbiome of preterm infants,^2^ individual’s identification (unique record number (URN)) was included as a random factor. To assess the influence of clinical variables at both admission and discharge, an interaction variable was included in the model (labelled Type). The resulting model, *Shannon* *~* *(15 Parameters)* * *Type* + *(1* | *URN)*, assesses the effect of the 15 predictors on Shannon diversity for both types of samples, Admission and Discharge, whilst accounting for the individual, represented here by URN.

Backward selection (69) was then implemented to simplify the model by comparing Akaike’s information criterion scores between regression models and removing predictors that were not contributing to the model. The process was repeated until the least complex adequate model was identified. The covariates included in the final model were sepsis, antenatal antibiotics, gestational age at birth, gestational age at collection, diet, the mode of delivery, NEC, preeclampsia, ROP and days on antibiotics. The significance of the fixed-effects variables in this final model was then assessed using: analysis of deviance (Type II Wald *χ*^2^ test) from the *car* package^61^ and post-hoc pairwise Tukey’s comparisons (correcting for multiple comparisons) from the *emmeans* package.^62^

For differential taxonomic abundance, two negative binomial generalised linear models were created using the package DESeq2. A combination of previous literature and exploratory analysis, including PCoA plots, PCA and scatterplots, were used for model selection. Again, continuous predictors were scaled and centered, and multicollinearity was assessed. Taxa were agglomerated at the genus level, due to the limited taxonomic depth of short amplicon sequencing. To reduce the number of false positives, two separate models were run; one each for admission and discharge samples. The resulting model assessed the effect of 11 independent predictors, sepsis, diet, chorioamnionitis, mode of delivery, gestation at birth, gestation at collection, NEC, preeclampsia, ROP and days on antibiotics prior to sample collection, on taxonomic abundance. Low abundance and low-frequency taxa were then removed, and a Wald test with the Benjamin–Hochberg multiple inference corrections was then performed. More information on the analysis can be found in the Supplementary Material.

## Results

### Exploring changes in composition and diversity from admission to discharge

**Fig. 1:**
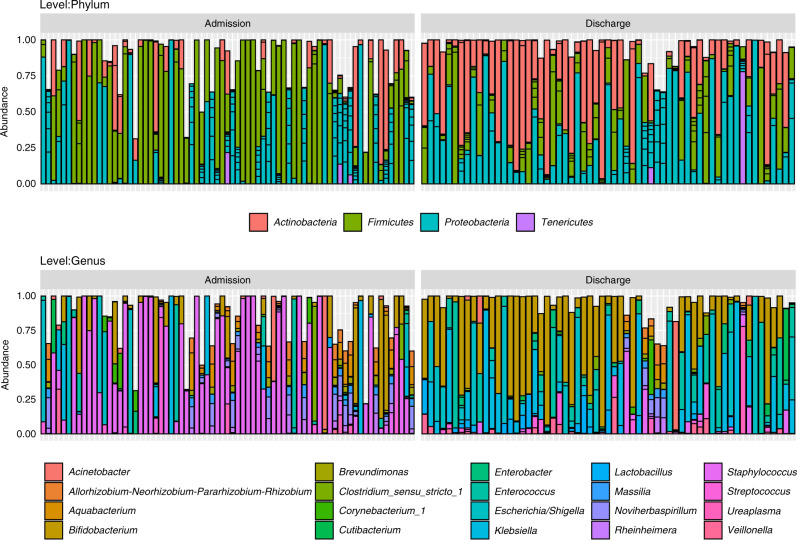
The distribution of the top 20 most abundant taxa in probiotic-treated, preterm infants. Histograms representing the distribution (top 20 taxa) of taxonomic relative abundance for admission and discharge samples at both phylum and genus levels.

**Fig. 2:**
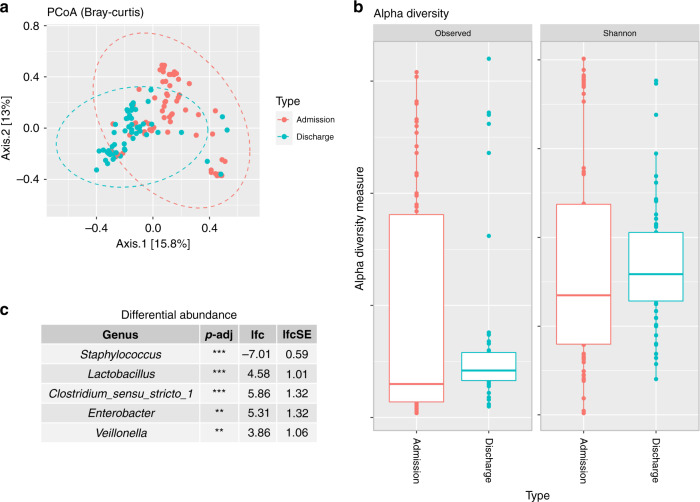
Univariate analyses exploring changes in the gut microbiome of probiotic-treated, preterm infants between admission and discharge. **a** Principal coordinate analysis plot for admission versus discharge based on Bray–Curtis dissimilarity matrix (*p* < 0.01 and *R*^2^ = 0.06), **b** box plots of alpha diversity for admission versus discharge and **c** table of differential abundance testing for admission versus discharge (base value is admission). p-adj Adjusted *p* value, lfc log-fold change, lfcSE log-fold change standard error. **p < 0.01 and ***p < 0.001.

The study recruited 85 preterm infants born <32 weeks and <1500 g from the THHS NICU. From these infants, 134 stool samples were collected, of which 71 were from admission (meconium) and 63 from discharge (stool). Other cohort demographics can be observed in Table 1. Significant changes in genera were observed between admission and discharge (Fig. 1), with *Staphylococcus* significantly higher at admission (*p* < 0.01), and *Enterobacter* (*p* < 0.01), *Lactobacillus* (*p* < 0.01), *Clostridium sensu stricto 1* (*p* < 0.01) and *Veillonella* (*p* < 0.05) higher at discharge (Fig. 2c). Although there was a limited separation between admission and discharge samples, the beta diversity showed a clustering pattern that resulted in a significant difference between the two groups (Fig. 2a, PERMANOVA; *p* < 0.01 and *R*^2^ = 0.06, homogeneity of variance; *p* = 0.85). The average species diversity within samples (Observed and Shannon) increased from admission to discharge (Fig. 2b), but not significantly.

### Exploring the effect of clinical variables on alpha diversity and taxonomic abundance

**Fig. 3:**
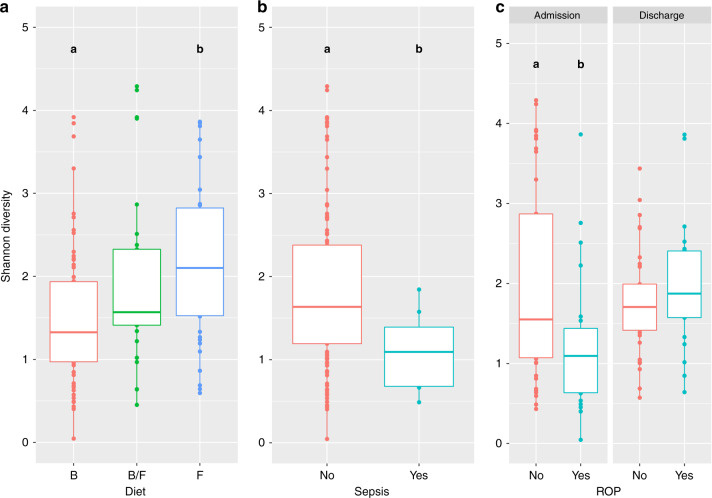
A comparison of alpha diversity between different levels of significant covariates of the probiotic-treated, preterm infant gut. Boxplots of alpha diversity (Shannon index) for significant Tukey’s pairwise comparisons designated by lower case letters (where a is significantly different from b) on linear mixed-effects model. Annotation for Diet: B breastmilk, B/F breastmilk and formula and F Formula. **a** Box plot comparing alpha diversity at admission and discharge between different types of diet, **b** box plot comparing alpha diversity between sepsis diagnoses and **c** box plot comparing alpha diversity at admission and discharge between retinopathy of prematurity diagnoses.

Several maternal and infant variables were significantly associated with changes seen in the preterm infant gut microbiome. Mixed-effects models show that several clinical and environmental variables were significantly associated with both the diversity and taxonomic composition within samples. Significant pairwise differences in diversity were observed for diet, sepsis and ROP (Fig. 3), and chorioamnionitis, preeclampsia, sepsis, NEC, ROP and diet were all associated with changes in taxonomy (Table 2).

**Table 2:** The significant differentially abundant taxa at the genus level obtained from DESeq2 analysis, with log 2 fold change for the variable listed compared to the base value.

Log 2 fold change	lfcSE	*p*_adj_	Genus	Variable	Sample
3.09	0.99	*	*Staphylococcus*	Chorioamnionitis: yes	Admission
−17.58	3.22	***	*Enhydrobacter*	Sepsis: yes	Admission
−15.38	3.95	**	*Pseudomonas*	Sepsis: yes	Admission
10.33	2.84	**	*Bifidobacterium*	Sepsis: yes	Admission
−11.62	2.31	***	*Bifidobacterium*	NEC: yes	Admission
4.84	1.00	***	*Staphylococcus*	ROP: yes	Admission
−27.65	2.64	***	*Escherichia*/*Shigella*	Preeclampsia: yes	Discharge
−4.25	1.67	*	*Veillonella*	Diet: breastmilk	Discharge
2.54	0.87	*	*Bifidobacterium*	Diet: breastmilk	Discharge
3.64	1.46	*	*Klebsiella*	Diet: breastmilk	Discharge
−5.52	1.91	*	*Lactobacillus*	Diet: formula	Discharge

*p-adj* adjusted *p* value, *lfc* log-fold change, *lfcSE* log-fold change standard error, *NEC* necrotising enterocolitis, *ROP* retinopathy of prematurity.

*P < 0.05, **p < 0.01 and ***p < 0.001 .

#### Mode of delivery and diet

Only diet had a significant impact on the gut microbiome, with the mode of delivery not reaching significance for alpha diversity (*p* = 0.057, Supplementary Material) or any taxa. The type of milk the infant received had a significant effect on alpha diversity (Fig. 3a; *χ*^2^ = 13.5, d.f. = 2, *p* < 0.01), with subsequent post-hoc pairwise comparisons finding a significant difference between formula-fed infants ($\bar x$ = 2.10 ± 0.17) and those that were breastfed ($\bar x$ = 1.56 ± 0.11) (Fig. 3a; *p* < 0.01). For differential abundance, infants who were fed only breastmilk had significantly higher abundances of both *Bifidobacterium* (Table 2; *p* < 0.05) and *Klebsiella* (Table 2; *p* < 0.05), and lower *Veillonella* (Table 2; *p* < 0.05), relative to those that were only fed formula, but only at discharge. In addition, those fed only formula had significantly lower *Lactobacillus* at discharge (Table 2; *p* < 0.01).

#### Pregnancy complications

Both preeclampsia and chorioamnionitis had a significant impact on the infant gut microbiome, with both conditions significantly influencing taxonomy (Table 2). In infants whose mothers were diagnosed with chorioamnionitis before or during labour, *Staphylococcus* was significantly higher at admission (Table 2; *p* < 0.05). For infants whose mother was diagnosed with preeclampsia, there were no differences at admission, but significantly lower *Escherichia*/*Shigella* (Table 2; *p* < 0.001) at discharge.

#### Neonatal complications

Three neonatal complications, ROP, NEC and sepsis, were found to significantly impact the developing preterm gut microbiome. Both sepsis (Fig. 3b; *χ*^2^ = 4.70, d.f. = 1, *p* < 0.05) and ROP (Fig. 3c; *χ*^2^ = 10.98, d.f. = 1, *p* = <0.001) significantly influenced diversity, with infants who were diagnosed with sepsis having significantly lower diversity ($\bar x$ = 1.10 ± 0.17) than infants who did not have the disease ($\bar x$ = 1.84 ± 0.09). For ROP, subsequent post-hoc analysis found pairwise differences at admission between infants who were diagnosed with the disease ($\bar x$ = 1.25 ± 0.18) and those who did not have ROP ($\bar x$ = 2.04 ± 0.18) (Fig. 3c; *p* < 0.01).

NEC, sepsis and ROP significantly influenced the abundances of several taxa at admission. Infants diagnosed with sepsis had significantly lower *Pseudomonas* (*p* < 0.01) and *Enhydrobacter* (*p* < 0.01), in combination with significantly enriched *Bifidobacterium* (*p* < 0.01). *Bifidobacterium* was significantly lower in infants diagnosed with NEC (*p* < 0.01), and *Staphylococcus* significantly enriched in infants diagnosed with ROP (*p* < 0.01).

## Discussion

The aim of this study was to identify and understand variation in gut microflora development in a cohort of probiotic-treated preterm infants from North Queensland, Australia. Specifically, we set out to assess the difference in bacterial microbiome between two time points while the infant was in hospital admission and discharge. We also sought to understand the effect of several clinical variables (both maternal and infant) on the development of the gut microbiome. To do so, we utilised 16S rRNA gene high-throughput sequencing. We then conducted univariate comparisons to examine the difference between the infant microbiome at admission and discharge, and mixed-effects models to explore the influence of several clinical variables, including Sepsis, Feeding Type, Chorioamnionitis, Mode of Delivery, Gestation, NEC, Preeclampsia and ROP.

### Exploring changes in composition and diversity from admission to discharge

Despite the overlap, the overall community structure was significantly different between admission and discharge faecal samples. *Staphylococcus*, commonly an early coloniser of the infant gut,^63^ was found in significantly higher abundance at admission. In healthy newborns, colonisation usually begins with oxygen-tolerant microbes^63^ like *Staphylococcus*, which consume oxygen, shifting the environment from aerobic to anaerobic,^64^ allowing colonisation of strict anaerobes.^63^ *Clostridium sensu stricto 1*, a genus of mostly strict anaerobes, along with the genera *Lactobacillus*, *Enterobacter* and *Veillonella* were found in significantly higher abundance at discharge. The significant presence of *Lactobacillus* at admission is surprising considering the delayed or limited colonisation of common commensals with *Lactobacillus* and *Bifidobacterium* normally seen in preterm infants.^10,15,65^ Although not significant (*p* = 0.11), the presence of *Bifidobacterium* across 99 samples in such a young cohort is also noteworthy.^65,66^ This is especially true considering their treatment with Infloran®, which may explain the significant presence of both *Lactobacillus* and *Bifidobacterium* in such a cohort. Future work should apply a more robust sequencing method to see if the species present are those found within the probiotic.

### Exploring the effect of clinical variables on alpha diversity and taxonomic abundance

#### Mode of delivery and diet

In contrast to previous studies, we observed no significant pairwise differences in diversity or taxonomy between vaginally and caesarean delivered infants at admission or discharge. Typically, caesarean-born infants bypass the vaginal route of inoculation, resulting in greater diversity,^16^ with fewer or delayed colonisation of *Lactobacillus*,^15^ *Bifidobacterium*^10,15^ and *Bacteroides*,^67–69^ coupled with higher than normal amounts of skin-dwelling microbes. The inconsistency between our results and the literature may be due to other confounding variables, such as prematurity itself or supplementation with probiotics, which has been demonstrated to alter *Bifidobacterium* and *Lactobacillus* populations in preterm infants.^37^ If probiotic treatment is driving the disparity between our results and previous work, this would support previous work suggesting probiotic supplementation can correct for microbial differences seen in caesarean-born infants.^70^

Regarding the influence of diet on the microbiome, we observed significantly lower alpha diversity and higher abundances of *Bifidobacterium* and *Klebsiella* at discharge in breastfed infants, relative to those solely formula-fed. The significant difference in *Bifidobacterium* supports previous work showing that breastfed infants have lower diversity^18^ in combination with more commensal microbes,^15,71^ including different *Bifidobacterium* species.^15^ The higher abundance of such microbes stems from the presence of both *Bifidobacterium* and HMOs in breastmilk.^72–74^ In addition, the higher abundance of Lactobacillus in infants who were fed a combination of formula and breastmilk, relative to those who only received formula, suggests that ‘supplementing’ formula feeding with some breastmilk may correct for some microbial imbalances associated with formula feeding. As for the differences in *Klebsiella*, the genus contains known pathogens such as *Klebsiella pneumoniae*, previously associated with NEC,^75^ and has been implicated in cases of sepsis. However, *K. pneumoniae* is a very diverse genus that is also part of normal flora.

#### Pregnancy complications

Maternal factors also significantly impacted the composition of the probiotic-treated preterm infant microbiome, with associations observed for both chorioamnionitis and preeclampsia. Infants whose mothers were diagnosed with chorioamnionitis had higher abundances of the genus *Staphylococcus*. Previous work has found microbes at different levels of taxonomy to be associated with chorioamnionitis, but not from the genus we observed.^76^ As chorioamnionitis is a bacterial infection of the placenta and membrane surrounding the foetus, occurring before or during labour, what pathogens are translocated from the membrane to the foetus may dictate the associations found. Unfortunately, the translocation and resulting increased abundance of *Staphylococcus* may be why exposure to chorioamnionitis increases the risk of preterm infants to adverse neonatal outcomes,^76^ like sepsis, which has previously been associated with *Staphylococcus*.^77,78^

For infants whose mothers were diagnosed with preeclampsia, *Escherichia*/*Shigella* was significantly lower at discharge. As preeclampsia can alter the maternal microbiome,^23^ the resulting dysbiosis, at least in part, may be being passed through a maternal route of inoculation. Previous work by Stewart et al., *The Environmental Determinants of Diabetes in the Young (TEDDY)* study, has found that preeclampsia contributes to significant differences at the species, but not genus, level.^9^ However, the two cohorts are vastly different, with the TEDDY study including both full- and preterm children, with samples from 3 months of age. In contrast, our cohort was entirely preterm, who at discharge may have only been 3 months old. In addition, as preeclampsia is associated with preterm birth,^79^ our cohort had a larger proportion of infants born to preeclamptic mothers (18% compared to 4%). Taken together, preeclampsia may have a greater impact on preterm infants, or may have more of an effect in the early months of life, when the mother is still the dominant colonising route for microbes. As to why Preeclampsia only has an effect at discharge is unclear. However, as the impact is occurring via the maternal route, it may be related to continued exposure to the mother. This continued exposure through the maternal route of transmission, either by touch or breastmilk, may compound the passing of irregular taxonomic profiles that resulting from continued preeclampsia treatment post delivery.^80,81^

#### Neonatal complications

We found that sepsis significantly influenced the abundance of *Bifidobacterium*, *Pseudomonas* and *Enhydrobacter*. Multi-omics approaches have previously linked sepsis to the gut microbiome,^82^ with other studies showing associations of sepsis with low diversity,^13^ as well as higher abundances of *Staphylococcus*,^77,78^ and lower abundances or absence of commensal microbes like *Bifidobacterium*.^31,82^ Although we also observed differences in *Bifidobacterium*, the directional effect is counter to what was observed previously. However, it is worth noting that of the eight infants diagnosed with sepsis, only three had *Bifidobacterium* in their sample. So, despite reaching statistical significance, this finding may not be clinically relevant.

For NEC, we observed significantly lower abundances of *Bifidobacterium*, but in contrast to previous work, no enrichment of any taxa. As previously mentioned, *Bifidobacterium* is a common commensal microbe found in the probiotic Infloran®, which is uncommon in preterm infants born <33 weeks gestation^65^ and has previously been shown to be protective against NEC.^83^ Although our work does not support previous evidence of an associated pathogen, the plethora of microbes that have previously been associated with NEC,^32,75,84^ in combination with studies showing reduced commensal microbes^85,86^ and diversity,^87,88^ suggests the aetiology is more complex than just the presence of a pathogen.

We also observed significant enrichment of *Staphylococcus* (of the *Staphylococcaceae* family) and lower diversity at admission for infants diagnosed with ROP. An association between the gut microbiota and ROP has been explored once before, by Skondra et al.^89^ They observed significant enrichment of the family *Enterobacteriaceae* in preterm infants with the disease at 28 weeks postmenstrual age.^89^ The discrepancy in our results is not necessarily a product of error, but rather, as seen with NEC, due to the complex aetiology characterised by more than just the presence of a particular group of taxa. This complexity makes it difficult to hypothesise the specific role that the microbiome could be playing in ROP. However, if a role is established there is potential for the microbiome to become a target for intervention, and thus this should be the target of further research.

Limitations of our work include low taxonomic depth and only sampling in early infancy. The use of 16S rRNA gene metabarcoding limited detection power to the genus level, resulting in no identification of species or functional genes. In addition, collecting samples only at admission and discharge means we have no insight into the longevity of the differences observed; this may impact the clinical significance. Future work will use a combination of 16S rRNA gene metabarcoding and shotgun metagenomic techniques to both characterise species allowing an exploration of the differences observed in this study and others, and to investigate if these persist in the long term.

This prospective observational study used 16S rRNA gene sequencing to characterise the bacterial microbiome of probiotic-supplemented infants. The study aimed to identify and understand variation in bacterial gut flora between two time points and as the result of several clinical variables. Our study builds on previous research and supports other studies describing significant changes in the preterm microbiome over time and associations with several factors. The lower bacterial diversity seen in infants diagnosed with diseases or who were formula-fed, as well as the differing abundances of several taxa across multiple variables, reinforces the role of the microbiome in disease and supports the need for promoting healthy microbiome development. In addition, the associations with maternal disease highlight the importance of maternal health to infant microbiome development, and in turn infant health.

**Supplementary information** All additional materials can be found at: https://github.com/JacobAFW/NICU_Microbiome_Study.

## Data Availability

The sequencing dataset generated and/or analysed during the current study is available through the International Nucleotide Sequence Database Collaboration at the National Center for Biotechnology Information (NCBI) repository, https://www.ncbi.nlm.nih.gov/bioproject/687291. BioProject ID: PRJNA687291.

## References

[1] Shreiner AB, Kao JY, Young VB (2015). The gut microbiome in health and in disease. Curr. Opin. Gastroenterol..

[2] Hill CJ (2017). Evolution of gut microbiota composition from birth to 24 weeks in the Infantmet Cohort. Microbiome.

[3] Dahl C (2018). Preterm infants have distinct microbiomes not explained by mode of delivery, breastfeeding duration or antibiotic exposure. Int. J. Epidemiol..

[4] Barrett E (2013). The individual-specific and diverse nature of the preterm infant microbiota. Arch. Dis. Child Fetal Neonatal Ed..

[5] Magne F (2006). Low species diversity and high interindividual variability in faeces of preterm infants as revealed by sequences of 16s rRNA genes and PCR-temporal temperature gradient gel electrophoresis profiles. FEMS Microbiol. Ecol..

[6] Arboleya S (2012). Establishment and development of intestinal microbiota in preterm neonates. FEMS Microbiol. Ecol..

[7] Chang JY, Shin SM, Chun J, Lee JH, Seo JK (2011). Pyrosequencing-based molecular monitoring of the intestinal bacterial colonization in preterm infants. J. Pediatr. Gastroenterol. Nutr..

[8] Schwiertz A (2003). Development of the intestinal bacterial composition in hospitalized preterm infants in comparison with breast-fed, full-term infants. Pediatr. Res..

[9] Stewart CJ (2018). Temporal development of the gut microbiome in early childhood from the Teddy Study. Nature.

[10] Grześkowiak Ł (2015). Gut Bifidobacterium microbiota in one-month-old Brazilian newborns. Anaerobe.

[11] Stewart CJ (2015). Preterm gut microbiota and metabolome following discharge from intensive care. Sci. Rep..

[12] Gomez, M. et al. Bacteriological and immunological profiling of meconium and fecal samples from preterm infants: a two-year follow-up study. *Nutrients* **9**, 1293 (2017). 10.3390/nu9121293 PMC5748744 29186903

[13] Xiong W, Brown CT, Morowitz MJ, Banfield JF, Hettich RL (2017). Genome-resolved metaproteomic characterization of preterm infant gut microbiota development reveals species-specific metabolic shifts and variabilities during early life. Microbiome.

[14] La Rosa PS (2014). Patterned progression of bacterial populations in the premature infant gut. Proc. Natl Acad. Sci. USA.

[15] Itani T (2017). Establishment and development of the intestinal microbiota of preterm infants in a Lebanese Tertiary Hospital. Anaerobe.

[16] Chernikova DA (2016). Fetal exposures and perinatal influences on the stool microbiota of premature infants. J. Matern. Fetal Neonatal Med..

[17] Dominguez-Bello MG (2010). Delivery mode shapes the acquisition and structure of the initial microbiota across multiple body habitats in newborns. Proc. Natl Acad. Sci. USA.

[18] Mshvildadze M (2010). Intestinal microbial ecology in premature infants assessed with non-culture-based techniques. J. Pediatr..

[19] Underwood MA (2015). Human milk oligosaccharides in premature infants: absorption, excretion, and influence on the intestinal microbiota. Pediatr. Res..

[20] Ferretti P (2018). Mother-to-infant microbial transmission from different body sites shapes the developing infant gut microbiome. Cell Host Microbe.

[21] Lemas DJ (2016). Exploring the contribution of maternal antibiotics and breastfeeding to development of the infant microbiome and pediatric obesity. Semin. Fetal Neonatal Med..

[22] Qin J (2012). A metagenome-wide association study of gut microbiota in type 2 diabetes. Nature.

[23] Lv LJ (2019). Early-onset preeclampsia is associated with gut microbial alterations in antepartum and postpartum women. Front. Cell. Infect. Microbiol..

[24] Obermajer, T. et al. Microbes in infant gut development: placing abundance within environmental, clinical and growth parameters. *Sci Rep-Uk* **7**, 11230 (2017). 10.1038/s41598-017-10244-x PMC5593852 28894126

[25] Bizzarro MJ, Raskind C, Baltimore RS, Gallagher PG (2005). Seventy-five years of neonatal sepsis at Yale: 1928-2003. Pediatrics.

[26] Barron LK (2017). Independence of gut bacterial content and neonatal necrotizing enterocolitis severity. J. Pediatr. Surg..

[27] Kalliomaki M, Collado MC, Salminen S, Isolauri E (2008). Early differences in fecal microbiota composition in children may predict overweight. Am. J. Clin. Nutr..

[28] Dietert RR (2017). The microbiome-immune-host defense barrier complex (microimmunosome) and developmental programming of noncommunicable diseases. Reprod. Toxicol..

[29] Dinan, T. G. & Cryan, J. F. Gut instincts: microbiota as a key regulator of brain development, ageing and neurodegeneration. *J. Physiol*. **595**, 489–503 (2016). 10.1113/JP273106 PMC5233671 27641441

[30] Van Den Berg JP, Westerbeek EAM, Bröring-Starre T, Garssen J, Van Elburg RM (2016). Neurodevelopment of preterm infants at 24 months after neonatal supplementation of a prebiotic mix: a randomized trial. J. Pediatr. Gastroenterol. Nutr..

[31] Mai V (2013). Distortions in development of intestinal microbiota associated with late onset sepsis in preterm infants. PLoS ONE.

[32] Mai, V. et al. Fecal microbiota in premature infants prior to necrotizing enterocolitis. *PLoS ONE* **6**, 10.1371/journal.pone.0020647 (2011). 10.1371/journal.pone.0020647 PMC3108958 21674011

[33] Torrazza RM, Neu J (2013). The altered gut microbiome and necrotizing enterocolitis. Clin. Perinatol..

[34] Russell SL (2012). Early life antibiotic-driven changes in microbiota enhance susceptibility to allergic asthma. EMBO Rep..

[35] Soleimani F, Zaheri F, Abdi F (2014). Long-term neurodevelopmental outcomes after preterm birth. Iran. Red. Crescent Med. J..

[36] Dardas M (2014). The impact of postnatal antibiotics on the preterm intestinal microbiome. Pediatr. Res..

[37] Abdulkadir B (2016). Routine use of probiotics in preterm infants: longitudinal impact on the microbiome and metabolome. Neonatology.

[38] Underwood MA (2009). A randomized placebo-controlled comparison of 2 prebiotic/probiotic combinations in preterm infants: impact on weight gain, intestinal microbiota, and fecal short-chain fatty acids. J. Pediatr. Gastroenterol. Nutr..

[39] Chrzanowska-Liszewska D, Seliga-Siwecka J, Kornacka MK (2012). The effect of *Lactobacillus rhamnosus* GG supplemented enteral feeding on the microbiotic flora of preterm infants-double blinded randomized control trial. Early Hum. Dev..

[40] Sawh SC, Deshpande S, Jansen S, Reynaert CJ, Jones PM (2016). Prevention of necrotizing enterocolitis with probiotics: a systematic review and meta-analysis. PeerJ.

[41] Queensland Health. *The Health of Queenslanders 2018* (Queensland Health, 2018).

[42] Evidence Based Probiotics. Infloran. https://www.infloran.com.au/?gclid=CjwKCAiA-_L9BRBQEiwA-bm5fjBoxiUHkDF7r40k4SgIjF7M_MDTTVue4HDOB6QFbsX1XD_WgJICshoCPY8QAvD_BwE (2019).

[43] Carroll IM, Ringel-Kulka T, Siddle JP, Klaenhammer TR, Ringel Y (2012). Characterization of the fecal microbiota using high-throughput sequencing reveals a stable microbial community during storage. PLoS ONE.

[44] Fiedorová K (2019). The iImpact of DNA extraction methods on stool bacterial and fungal microbiota community recovery. Front. Microbiol..

[45] Tremblay, J. et al. Primer and platform effects on 16s rRNA tag sequencing. *Front. Microbiol.* **6**, 771 (2015). 10.3389/fmicb.2015.00771 PMC4523815 26300854

[46] Almeida, A., Mitchell, A. L., Tarkowska, A. & Finn, R. D. Benchmarking taxonomic assignments based on 16S rRNA gene profiling of the microbiota from commonly sampled environments. *Gigascience* **7**, giy054 (2018). 10.1093/gigascience/giy054 PMC5967554 29762668

[47] Meridian. ISOLATE DNA KIT, Product Manual. *Meridian Bioscience*. https://www.bioline.com/ (2020).

[48] Illumina Inc. 16s Metagenomic sequencing library preparation. https://support.illumina.com/documents/documentation/chemistry_documentation/16s/16s-metagenomic-library-prep-guide-15044223-b.pdf.

[49] Illumina Inc. 16S Metagenomic Sequencing Library Preparation. *Illumina*. https://www.illumina.com/index-d.html (2020).

[50] Thermo Fisher Scientific. Platinum™ SuperFi™ PCR Master Mix. *Thermo Fisher Scientific*. https://www.google.com/search?q=platinum+superfi+pcr+master+mix&oq=platinum+superfi&aqs=chrome.1.69i57j0l7.3863j0j4&sourceid=chrome&ie=UTF-8 (2020).

[51] Rstudio. *Rstudio: Integrated Development for R* (PBC, 2020).

[52] Callahan BJ, Sankaran K, Fukuyama JA, McMurdie PJ, Holmes SP (2016). Bioconductor workflow for microbiome data analysis: from raw reads to community analyses. F1000Research.

[53] Callahan BJ (2016). Dada2: high-resolution sample inference from Illumina Amplicon Data. Nat. Methods.

[54] McKnight D (2019). Microdecon: a highly accurate read‐subtraction tool for the post‐sequencing removal of contamination in metabarcoding studies. Environ. DNA.

[55] McMurdie PJ, Holmes S (2013). Phyloseq: an R package for reproducible interactive analysis and graphics of microbiome census data. PLoS ONE.

[56] Oksanen Jari, MASS Suggests. (2007). The Vegan package. Community Ecol. Package.

[57] Benjamini Y, Hochberg Y (1995). Controlling the false discovery rate: a practical and powerful approach to multiple testing. J. R. Stat. Soc. Ser. B.

[58] Love MI, Huber W, Anders S (2014). Moderated estimation of fold change and dispersion for RNA-Seq Data with Deseq2. Genome Biol..

[59] Bates, D., Mächler, M., Bolker, B. & Walker, S. Fitting linear mixed-effects models using Lme4. *J. Stat. Softw.* **67**, 48 (2015).

[60] AED. *Package Accompanying ‘Mixed Effects Models and Extensions in Ecology with R’* (Springer, 2009).

[61] Fox, J. & Weisberg, S. *An {R} Companion to Applied Regression* 3rd edn (Sage, 2019).

[62] Searle SR, Speed FM, Milliken GA (1980). Population marginal means in the linear model: an alternative to least squares means. Am. Statistician.

[63] Mackie RI, Sghir A, Gaskins HR (1999). Developmental microbial ecology of the neonatal gastrointestinal tract. Am. J. Clin. Nutr..

[64] Jost T, Lacroix C, Braegger CP, Chassard C (2012). New insights in gut microbiota establishment in healthy breast fed neonates. PLoS ONE.

[65] Butel MJ (2007). Conditions of bifidobacterial colonization in preterm infants: a prospective analysis. J. Pediatr. Gastroenterol. Nutr..

[66] Campeotto F (2011). A fermented formula in pre-term infants: clinical tolerance, gut microbiota, down-regulation of faecal calprotectin and up-regulation of faecal secretory Iga. Br. J. Nutr..

[67] Gregory KE (2016). Influence of maternal breast milk ingestion on acquisition of the intestinal. Microbiome Preterm Infants Microbiome.

[68] Arboleya S (2015). Intestinal microbiota development in preterm neonates and effect of perinatal antibiotics. J. Pediatr..

[69] Bennet R, Nord CE (1987). Development of the faecal anaerobic microflora after caesarean section and treatment with antibiotics in newborn infants. Infection.

[70] Korpela K (2018). Probiotic supplementation restores normal microbiota composition and function in antibiotic-treated and in caesarean-born infants. Microbiome.

[71] Mastromarino P (2014). Correlation between Lactoferrin and beneficial microbiota in breast milk and infant’s feces. Biometals.

[72] Díaz-Ropero MP (2007). Two Lactobacillus strains, isolated from breast milk, differently modulate the immune response. J. Appl. Microbiol..

[73] Martín R (2009). Isolation of bifidobacteria from breast milk and assessment of the bifidobacterial population by PCR-denaturing gradient gel electrophoresis and quantitative real-time PCR. Appl. Environ. Microbiol..

[74] Bode L (2009). Human milk oligosaccharides: prebiotics and beyond. Nutr. Rev..

[75] Sim K (2015). Dysbiosis anticipating necrotizing enterocolitis in very premature infants. Clin. Infect. Dis..

[76] Puri K (2016). Association of chorioamnionitis with aberrant neonatal gut colonization and adverse clinical outcomes. PLoS ONE.

[77] Stewart CJ (2012). The preterm gut microbiota: changes associated with necrotizing enterocolitis and infection. Acta Paediatr..

[78] Madan JC (2012). Gut microbial colonisation in premature neonates predicts neonatal sepsis. Arch. Dis. Child Fetal Neonatal Ed..

[79] Davies EL, Bell JS, Bhattacharya S (2016). Preeclampsia and preterm delivery: a population-based case–control study. Hypertens. Pregnancy.

[80] Ishimwe JA (2021). Maternal microbiome in preeclampsia pathophysiology and implications on offspring health. Physiol. Rep..

[81] Choi MS, Yu JS, Yoo HH, Kim DH (2018). The role of gut microbiota in the pharmacokinetics of antihypertensive drugs. Pharmacol. Res..

[82] Stewart CJ (2017). Longitudinal development of the gut microbiome and metabolome in preterm neonates with late onset sepsis and healthy controls. Microbiome.

[83] Stewart CJ (2016). Temporal bacterial and metabolic development of the preterm gut reveals specific signatures in health and disease. Microbiome.

[84] Cassir N (2015). *Clostridium butyricum* strains and dysbiosis linked to necrotizing enterocolitis in preterm neonates. Clin. Infect. Dis..

[85] Pammi M (2017). Intestinal dysbiosis in preterm infants preceding necrotizing enterocolitis: a systematic review and meta-analysis. Microbiome.

[86] Warner BB (2016). Gut bacteria dysbiosis and necrotising enterocolitis in very low birthweight infants: a Prospective Case-Control Study. Lancet.

[87] McMurtry VE (2015). Bacterial diversity and clostridia abundance decrease with increasing severity of necrotizing enterocolitis. Microbiome.

[88] Wang Y (2009). 16S rRNA gene-based analysis of fecal microbiota from preterm infants with and without necrotizing enterocolitis. ISME J..

[89] Skondra, D. et al. The early gut microbiome could protect against severe retinopathy of prematurity. *J. AAPOS*, 10.1016/j.jaapos.2020.03.010 (2020). 10.1016/j.jaapos.2020.03.010 PMC7680397 32707176

