# Correction: The bacterial gut microbiome of probiotic-treated very-preterm infants: changes from admission to discharge

**DOI:** 10.1038/s41390-021-01888-7

**Published:** 2021-12-09

**Authors:** Jacob A. F. Westaway, Roger Huerlimann, Yoga Kandasamy, Catherine M. Miller, Robert Norton, Kyran M. Staunton, David Watson, Donna Rudd

**Affiliations:** 1 College of Public Health, Medical and Veterinary Science, James Cook University, 1/14-88 McGregor Road, Smithfield, QLD 4878 Australia; 2 Centre for Tropical Bioinformatics and Molecular Biology, James Cook University, 1 James Cook Drive, Douglas, QLD 4811 Australia; 3 Marine Climate Change Unit, Okinawa Institute of Science and Technology (OIST), 1919-1 Tancha, Onna-son Okinawa, 904-0495 Japan; 4 College of Science and Engineering, James Cook University, 1 James Cook Drive, Douglas, QLD 4811 Australia; 5 College of Public Health, Medical and Veterinary Science, James Cook University, 1 James Cook Drive, Douglas, QLD 4811 Australia; 6 Department of neonatology, Townsville University Hospital, 100 Angus Smith Drive, Douglas, QLD 4814 Australia; 7 Australian Institute for Tropical Health and Medicine, James Cook University, 1/14-88 McGregor Road, Smithfield, QLD 4878 Australia; 8 Department of Microbiology, Pathology Queensland, 100 Angus Smith Drive, Douglas, QLD 4814 Australia; 9 Department of Maternal-Fetal Medicine, Townsville University Hospital, 100 Angus Smith Drive, Douglas, 4814 Australia

Correction to: *Pediatric Research* 10.1038/s41390-021-01738-6, published online 7 October 2021

The article “The bacterial gut microbiome of probiotic-treated very-preterm infants: changes from admission to discharge”, written by Jacob A. F. Westaway, Roger Huerlimann, Yoga Kandasamy, Catherine M. Miller, Robert Norton, Kyran M. Staunton, David Watson, and Donna Rudd, was originally published electronically on the publisher’s internet portal on 7 October 2021 without open access. With the author(s)’ decision to opt for Open Choice the copyright of the article changed on 11 November 2021 to © The Author(s) 2021 and the article is forthwith distributed under a Creative Commons Attribution 4.0 International License, which permits use, sharing, adaptation, distribution and reproduction in any medium or format, as long as you give appropriate credit to the original author(s) and the source, provide a link to the Creative Commons licence, and indicate if changes were made.

The images or other third party material in this article are included in the article’s Creative Commons licence, unless indicated otherwise in a credit line to the material. If material is not included in the article’s Creative Commons licence and your intended use is not permitted by statutory regulation or exceeds the permitted use, you will need to obtain permission directly from the copyright holder.

